# Replacement of Metaphylactic Antimicrobial Therapy by Oral Administration of *Ligilactobacillus salivarius* MP100 in a Pig Farm

**DOI:** 10.3389/fvets.2021.666887

**Published:** 2021-05-31

**Authors:** Odón J. Sobrino, Claudio Alba, Rebeca Arroyo, Inés Pérez, Lydia Sariego, Susana Delgado, Leónides Fernández, Julián de María, Pilar Fumanal, Antonio Fumanal, Juan M. Rodríguez

**Affiliations:** ^1^Scientific Society of Veterinary Public and Community Health (SOCIVESC), Madrid, Spain; ^2^Department of Nutrition and Food Science, Complutense University of Madrid, Madrid, Spain; ^3^Department of Microbiology and Biochemistry, Dairy Research Institute of Asturias, Villaviciosa, Spain; ^4^Department of Galenic Pharmacy and Food Technology, Complutense University of Madrid, Madrid, Spain; ^5^L'Albeitar, Guaso, Huesca, Spain

**Keywords:** swine, antimicrobials, antibiotic resistance, probiotics, *Ligilactobacillus salivarius*, short chain fatty acids, microbiome

## Abstract

Antibiotic use in swine production contributes to the emergence and spread of resistant bacteria, which poses a threat on human health. Therefore, alternative approaches must be developed. The objective of this work was the characterization of the probiotic properties of a *Ligilactobacillus salivarius* strain isolated from sow's milk and its application as an inoculated fermented feed to pregnant sows and piglets. The study was carried in a farm in which metaphylactic use of antimicrobials (including zinc oxide) was eliminated at the time of starting the probiotic intervention, which lasted for 2 years. Feces from 8-week-old piglets were collected before and after the treatment and microbiological and biochemical analyses were performed. The procedure led to an increase in the concentrations of clostridia and lactobacilli-related bacteria. Parallel, an increase in the concentration of butyrate, propionate and acetate was observed and a notable reduction in the presence of antibiotic resistant lactobacilli became apparent. In conclusion, replacement of antimicrobials by a microbiota-friendly approach was feasible and led to positive microbiological and biochemical changes in the enteric environment.

## Introduction

During the last decades, antibiotic-(multi)resistant bacteria have become a global threat for human health. The overuse, abuse and misuse of antibiotics in humans and animals have accelerated the development and spread of resistances. It has been suggested that the current “antibiotic resistance crisis” may lead us back to a “pre-antibiotic era” if effective actions to significantly decrease antibiotic use are not undertaken rapidly (1–4).

Prolonged prophylactic and metaphylactic use of antibiotics is widespread in intensive food animal management systems worldwide as low-cost growth promoters (5). The addition of sub-therapeutic levels of antibiotics in feed or water can improve growth rates by reducing the morbidity and mortality burden associated to bacterial diseases (6). However, such practice has notably contributed to the emergence and spread of resistant bacteria, both by direct contact with antibiotic-resistant bacteria from livestock or by indirect contact through food, water, and animal waste (7, 8). Swine production is responsible for a high proportion of the antimicrobials used in food animal production (6). Despite of the EU ban regarding the use of antibiotics as growth promoters, these antimicrobials have been frequently employed in the last years in swine farming as either prophylactic or metaphylactic agents. The spread of resistance to antibiotics will probably limit the therapeutic choices and increase morbidity and mortality rates due to porcine or human infections caused by resistant bacteria (3). A worrying example was the emergence of an *Escherichia coli* strain carrying a plasmid-mediated colistin-resistance gene in both people and pigs in China (9) and its rapid spread to these and other host species in Europe and North America (10–13). In addition to antibiotics, zinc oxide is another antimicrobial substance generally employed in swine production to try to prevent or minimize post-weaning diarrhea. Several alternatives have been suggested in order to replace routine use of prophylactic or metaphylactic antimicrobials in pig production, including acidifiers, prebiotics and probiotics.

In this context, the objectives of this work were, first, to isolate and characterize a probiotic candidate from milk of an in-house sow with a good record of reproductive outcomes; second, to apply the strain as strategy to replace routine metaphylactic use of antibiotics in the farm where the strain was isolated; third, to evaluate the microbiological and biochemical impact of that replacement strategy.

## Materials and Methods

### Isolation and Identification of *L. salivarius* MP100 From Porcine Milk

Milk was collected as previously described (14) at day 14 after delivery from a healthy 7-years-old in-house sow with a good record of reproductive outcomes. The sample was kept frozen (−20°C) until delivery to the laboratory. The sample was diluted in peptone water, 100 μL of the dilutions were spread on Man, Rogosa, and Sharpe (MRS; Oxoid, Basingstoke, United Kingdom) agar plates supplemented with L-cysteine (0.5 g/L) (MRS-Cys) and incubated aerobically at 37°C for 48 h. Only two colony morphologies were observed on the plates; one representative of each was selected and transferred to MRS broth tubes, which were incubated overnight under the same conditions without agitation. The isolate that reached the highest density (~9 log_10_ CFU/mL) was identified as *L. salivarius* by 16S rRNA gene sequencing following the procedure described by Kullen et al. (15).

### Survival of *L. salivarius* MP100 After Exposition to Conditions Similar to Those of the Porcine GIT

*L. salivarius* MP100 was tested using portions of a commercial antibiotic-free swine feed (50 g) containing ~10^9^ CFU/mL of the strain in an *in vitro* model simulating passage through the oral cavity, the stomach and the small intestine, as described by Marteau et al. (16) with the modifications included by Martín et al. (17), using porcine gastric juice (5 mL; chloride: 129 mmol/L; sodium: 68 mmol/L; pH 3.4) obtained in an abattoir (Madrid Norte, San Agustín de Guadalix, Madrid, Spain). The pH curve in the stomach-resembling compartment was controlled as described for monogastric mammals (18). After 120 min of total exposition, bacterial survival was determined by plating the samples onto MRS agar plates, which were anaerobically incubated at 37°C for 48 h. *Lacticaseibacillus rhamnosus* GG, a well-characterized probiotic strain was used as a control because of its high survival rate in the same *in vitro* model (17).

### Determination of the Antimicrobial Activity and Antimicrobial Compounds Produced by *L. salivarius* MP100

An overlay method (19) was used to determine the ability of *L. salivarius* MP100 to inhibit the growth of various bacterial species. The strain was inoculated (~2-cm-long lines) on MRS agar plates and incubated at 32°C for 48 h in anaerobic jars (Oxoid). Then, the indicator microorganisms (~10^4^ CFU) vehiculated in 10 mL of soft (0.7% agar) BHI (Oxoid) were inoculated on top. The bacteria employed as indicator organisms (our own culture collection) were originally isolated from feces of pigs or piglets with gastroenteritis (mainly diarrhea), septicemia, arthritis or meningitis symptoms, and included *Clostridium perfringens* MP34, *Enterococcus faecalis* MP42, *Staphylococcus aureus* MP83, *Streptococcus suis* MP205, *Trueperella pyogenes* MP214, *Escherichia coli* MP73 (F4) and MP77 (F18), *Salmonella enterica* serovar Typhimurium MP55, and *Klebsiella pneumoniae* MP87. The plates overlaid with bacterial indicators were further incubated according to the optimal growth temperature of the indicator microorganism (32 or 37°C) for 48 h. Finally, the clear zones of inhibition (>2 mm) around the strain streaks were measured. All experiments assaying inhibitory activity were performed in triplicate.

Subsequently, the concentrations of L- and D-lactic acid in the supernatants obtained from MRS cultures (incubated for 16 h at 37°C) of *L. salivarius* MP100 were determined with an enzymatic kit (Roche Diagnostics, Mannheim, Germany), following the manufacturer's instructions. The pH values of the supernatants were also measured. The ability of *L. salivarius* MP100 to produce bacteriocins was determined in culture supernatants by the agar well diffusion assay described by Dodd et al. (20) and modified by Martín et al. (21), using as indicator bacteria the Gram-positive strains employed for the overlay method. The Gram-positive strains listed above for the initial screening for antimicrobial activity were employed as indicators of bacteriocinogenic activity. Since *L. salivarius* MP100 showed bacteriocinogenic activity against some of the indicators, PCR assays were performed to detect the structural genes corresponding to the *L. salivarius* bacteriocins salivaricin B and bacteriocin Abp-118 produced by this bacterial species, following the procedures described by Çataloluk (22) and Flynn et al. (23), respectively.

### Adherence to Epithelial Cells and Porcine Mucin

The adherence of *L. salivarius* MP100 to HT-29 and Caco-2 cells was examined as described by Coconnier et al. (24). The adhesion of bacterial cells of this strain to porcine mucin was determined according to the procedure reported by Cohen and Laux (25) and the modifications of Olivares et al. (26). The assays were performed in triplicate and the values were expressed as the mean (±SD) number of adherent cells in 20 random microscopic fields. *L. rhamnosus* GG was used as a control strain in these assays because of its high adherence to these epithelial cells and to porcine mucin (17).

### Safety-Related Characterization of *L. salivarius* MP100

The sensitivity of *L. salivarius* MP100 to antibiotics was determined by the E-test (BioMèrieux) using the cut-off levels established by EFSA for the antibiotics indicated for this species (27). The potential of *L. salivarius* MP100 to degrade partially purified porcine gastric mucin (HGM; Sigma) *in vitro* was evaluated following the plate procedure developed by Zhou et al. (28). Other safety-related analysis included the study of potential hemolysis using fresh horse blood agar plates (29), and the ability of the strain to produce biogenic amines (cadaverine, putrescine, histamine, and tyramine) from their respective precursor amino acids (30). These assays were performed in triplicate.

### Suppression of Metaphylactic Antibiotherapy in an Intensive Swine Farm and Replacement by Oral Administration of *L. salivarius* MP100

#### General Design and Sampling

The trial was conducted in an industrial closed cycle pig farm with a farrow-to-finish herd of 210 genetically similar Large White × Landrace sows. Sows, weaning piglets and fattening pigs were kept in high-investment indoor facilities following the UE standards requirements for animal welfare. Strict biosecurity protocols are followed on the farm, so that any animal or outsider is prevented from entering the production areas. Production farm management includes a 3-week batch system with an “all-in, all-out” procedure, with extensive cleaning followed by a sanitary break dry period of seven days between different batches. Piglets are weaned at 4 weeks of age. Until a few months before the starting of the assay, the farm had applied routine metaphylactic treatment during the perinatal period, consisting of the feed administration of zinc oxide, amoxicillin and colistin at the doses and for the periods of time prescribed by the veterinarian and recommended in the marketing authorization of authorized premixes for medicated feeding stuffs. Following the recommendations of the health authorities, the use of antimicrobials was eliminated gradually, starting with colistin and finishing with zinc oxide. The assay described below started once all the antimicrobials had been withdrawn.

From day 0 (sampling time T1), the diet of the animals was exactly the same that they were receiving before with the only exception that all antimicrobials (including zinc oxide) were completely removed from the feed. In addition, the strain *L. salivarius* MP100 was orally administered (~9 log_10_ CFU daily) to pregnant sows (during the week previous to farrowing and throughout the lactation period) and, also, to piglets continuously from 12 days after birth to the start of the fattening stage, through an inoculated fermented feed (IFF). This specific strain dose was selected because it has been shown to be efficient to modulate the host microbiota in previous clinical trials involving other *L. salivarius* strains (31, 32). This probiotic supplementation was carried out continuously in the farm for 2 years (sampling time T2). No control or placebo batches were included during the assay to avoid unintentionally transfer of the strain from treated to untreated animals. At sampling times T1 and T2, fecal samples from 15 different randomly-selected 8-week-old piglets were collected in sterile containers directly from the rectum with the aid of sterile gloves and spatula, aliquoted (2 aliquots of ~10 g), and stored at −20°C until processed as described below.

All animals were treated in strict accordance with the guidelines of the European Directive 2010/63/UE on the protection of animals used for scientific purposes. The study was approved by Ethical Committee on Animal Experimentation of the Faculty of Veterinary of the Universidad Complutense de Madrid (Spain), under protocol 33/17.

#### Analysis of SCFAs

Analysis of SCFAs (acetate, propionate, and butyrate) was performed using a dilution gas chromatography-mass spectrometry (GC-MS) assay as previously described (33, 34).

#### Assessment of the Fecal *Lactobacillus* Population by Culture-Dependent Methods

Fecal samples collected during the trial were serially diluted, plated onto MRS-Cys plates and incubated anaerobically (85% nitrogen, 10% hydrogen, 5% carbon dioxide) in an anaerobic workstation (DW Scientific, Shipley, UK) for up to 72 h at 37°C. After incubation, the number of colonies were recorded and at least one representative of each colony morphology was selected from the agar plates. The isolates were identified by Matrix Assisted Laser Desorption Ionization-Time of Flight (MALDI-TOF) mass spectrometry (Bruker, Germany). When the identification by MALDI-TOF was not possible at the species level (particularly in the case of lactobacilli isolates), the identification was carried out by 16S ribosomal RNA (rRNA) gene sequencing as described by Mediano et al. (35).

The isolates identified as *Limosilactobacillus reuteri, Lactobacillus johnsonii*, and *Lactobacillus amylovorus* were genotyped by RAPD profiling as described (36). The sensitivity of one representative of each different genotype to antibiotics was determined by the E-test (BioMèrieux) using the cut-off levels established by the EFSA for the antibiotics indicated for these species (27). Finally, a subset of 14 strains was assessed for the presence of genes conferring transmissible resistance to erythromycin (*ermB*) and tetracycline (*tetW* and *tetL*) as described in previous works (37, 38).

#### DNA Extraction From the Samples

Approximately 1 g of each fecal sample was used for DNA extraction following a method described previously (39). Extracted DNA was eluted in 22 μL of nuclease-free water and stored at −20°C until further analysis. Purity and concentration of each extracted DNA was initially estimated using a NanoDrop 1000 spectrophotometer (NanoDrop Technologies, Inc., Rockland, USA). Negative controls (blanks) were processed in parallel.

#### Real-Time Quantitative PCR Assays for the Specific Detection and Quantification of *L. salivarius* and *L. reuteri DNA*

Quantification of *L. salivarius* and *L. reuteri* DNA was carried using the procedures described by Harrow et al. (40) and Haarman and Knol (41), respectively. The DNA concentration of all samples was adjusted to 5 ng/μL. A commercial real-time PCR thermocycler (CFX96™, Biorad Laboratories, Hercules, CA, USA) was used for all experiments. Standard curves using 1:10 DNA dilutions (ranging from 2 ng to 0.2 pg) from *L. salivarius* CECT5713 and *L. reuteri* MP07 (our own collection) were used to calculate the concentrations of the unknown bacterial genomic targets. Threshold cycle (Ct) values between 15.29 and 20.07 were obtained for this range of bacterial DNA (*R*^2^ ≥ 0.992). The Ct values measured for DNA extracted from a non-target species (*Lactiplantibacillus plantarum* MP02; our own collection) was ≥39.27 ± 0.64. This control strain was selected because it is closely related, from a taxonomical point of view, to *L. salivarius* and *L. reuteri* (42). All samples and standards were run in triplicate.

#### Metataxonomic Analysis

The V3-V4 hypervariable region of the 16S rDNA was amplified by PCR using the universal primers S-D-Bact-0341-b-S-17 (CCTACGGGNGGCWGCAG) and S-D-Bact-129 0785-a-A-21 (GACTACHVGGGTATCTAATCC) (43) and sequenced in the MiSeq system of Illumina at the facilities of Parque Científico de Madrid (Tres Cantos, Spain). Barcodes appended to 3' and 5' terminal ends of the PCR amplicons allowed separation of forward and reverse sequences in a second PCR-reaction. DNA concentration of the PCR products was quantified in a 2100 Bioanalyzer system (Agilent, Santa Clara, CA, USA). After pooling the PCR products at about equal molar ratios, DNA amplicons were purified by using a QIAEX II Gel Extraction Kit (Qiagen) from the excised band having the correct size after running on an agarose gel. DNA concentration was then quantified with PicoGreen (BMG Labtech, Jena, Germany). The pooled, purified and barcoded DNA amplicons were sequenced using the Illumina MiSeq pair-end protocol (Illumina Inc., San Diego, CA, USA) following the manufacturer's protocols.

#### Bioinformatic Analysis

Raw sequence data were demultiplexed and quality filtered using Illumina MiSeq Reporter analysis software. Microbiome bioinformatics was done with QIIME 2 2019.1 (44). Denoising was performed with DADA2 (45). The forward reads were truncated at position 285 by trimming the last 15 nucleotides while the reverse ones were truncated at the 259 nucleotides by trimming the last 10 nucleotides. Taxonomy was assigned to amplicon sequence variants (ASVs) using the *q2-feature-classifier* (46) and the naïve Bayes classifier *classify-sklearn* against the SILVA database version 138 (47). Posterior bioinformatic analysis was conducted using the R version 3.5.1 (R Core Team, 2013; https://www.R-project.org). The *decontam* package was used in order to identify, visualize and remove contaminating DNA with the concentration of extracted DNA. The 5 most-abundant phyla and the 19 most abundant genera from all the milk samples were selected for comparison between groups of samples.

#### Statistical Analysis

The sample size required to detect a difference of 1 log_10_ CFU/g in the mean value of fecal lactobacilli counts between samples taken before (T1) and after (T2) the probiotic treatment in the pig farm and of 2 log_10_ CFU/g in the mean value of fecal lactobacilli counts between samples taken after the probiotic treatment in the pig farm (T2) and samples from the control farm (C; an intensive pig farm in which routine metaphylactic treatment was routinely used) was calculated using G^*^Power 3.1.5 (48). Preliminary data indicated a great variation in the fecal lactobacilli counts in pig fecal samples (1.25 log_10_ CFU/g) which, given the magnitude of the difference to detect, rendered an effect size of 0.63. The study would require 30 samples, equally distributed into two groups (T1 and T2), using a one-way ANOVA test at 5% level of significance and a statistical power of 95%.

Normally distributed data are reported as means and 95% confidence intervals (CI) or as means and standard deviations (SD), and non-normally distributed data by medians and interquartile ranges (IQR). Exploratory multifactorial or two-way ANOVA tests were performed to assess globally the impact of the supplementation with the probiotic strain. One-way ANOVA tests were used to compare the mean values of the different variables between the three groups of pigs. Tukey's HSD *post-hoc* tests were performed when required to identify which specific group's means were different after comparing all pairs of means. *t*-Tests allowed comparing the changes between the mean values of different parameters at the beginning and to the end of the probiotic assay. For non-normally distributed data, differences between groups were assessed using Kruskal-Wallis tests and pairwise Wilcoxon rank sum tests to compare data between farms. Bonferroni corrections were made to control for multiple comparisons. Statistical analysis and plotting were performed either using Statgraphics Centurion XVIII version 18.1.06 (Statgraphics Technologies, Inc., The Plains, VA, USA) or in the R environment and *ggplot2*. Differences were considered statistically significant at *p* < 0.05.

## Results

### Isolation and Identification of the Strain From the Porcine Milk Sample

Identification by 16S rRNA gene sequencing of the isolate that showed the best growth revealed that it belonged to the species *Ligilactobacillus salivarius* and the nomenclature MP100 was ascribed to the strain. This species was previously known as *Lactobacillus salivarius* but the name changed following the recent reclassification of the species within the genera *Lactobacillus* and *Leuconostoc* (49), and it is included in the QPS list of microorganisms with qualified presumption of safety of the European Food Safety Authority (EFSA) (50).

### Survival of *L. salivarius* MP100 After Exposition to Conditions Similar to Those Found in the Porcine Gastrointestinal Tract

*L. salivarius* MP100 showed a significant survival rate under simulated porcine GIT conditions. Exposure to a saliva-like solution had no negative effect on the strain while the survival rate after the transit through the stomach- and small intestine-like compartments was ~45% of the population initially inoculated. This value was comparable to that of the well-characterized probiotic strain *L. rhamnosus* GG (41%).

### Antimicrobial Activity of *L. salivarius* MP100

*L. salivarius* MP100 showed a noticeable inhibitory antimicrobial activity (inhibition zone > 0.5 cm around the streaks) against all indicator organisms used in this study (Table 1). This antibacterial effect was particularly effective against the Gram-negative indicator strains because of their sensitivity to the low pH of the *L. salivarius* MP100 supernatants, which is a result of the production of lactic acid. In fact, neutralization of the strain culture pH led to loss of the antimicrobial activity against the Gram-negative indicators while remaining unaffected for the Gram-positives. *L. salivarius* MP100 exclusively produced the L-lactic acid isomer, which reached a concentration of 10.29 (±0.31) mg/mL (pH 3.92) after growth in MRS broth for 16 h at 37°C. The production by *L. salivarius* CECT9145 [a high acidifying strain used as a control (16)] was 10.09 (±0.45) mg/mL (pH 3.97). *L. salivarius* MP100 showed bacteriocinogenic activity against some of the Gram-positive indicator bacteria (Table 1) although it did not harbor the structural genes encoding salivaricins B, OR-7 or Abp118. This suggests that this strain produces a novel bacteriocin.

**Table 1:** Antimicrobial activity of neutralized culture supernatants of *L. salivarius* MP100 (dimeter of the halos in cm).

**Indicator**	**Overlaid method**	**Well diffusion assay**
*Enterococcus faecalis* MP42	0.8	0.7
*Clostridium perfringens* MP34	1.9	1.8
*Staphylococcus aureus* MP83	2.1	2.2
*Streptococcus suis* MP205	0.7	0.6
*Trueperella pyogenes* MP214	1.1	1.0
*Escherichia coli* MP73 (F4)	2.9	Nd
*Escherichia coli* MP77 (F18)	3.1	Nd
*Salmonella enterica* serovar Typhimurium MP55	3.2	Nd
*Salmonella cholerasuis* CECT409	2.7	Nd
*Salmonella cholerasuis* CECT443	2.4	Nd
*Salmonella enteritidis* CECT4396	2.8	Nd
*Klebsiella pneumoniae* MP87	3.0	Nd
*Klebsiella pneumoniae* CECT 142	3.2	Nd

*Nd, not detected*.

### Adherence of *L. salivarius* MP100 to Epithelial Cells and Porcine Mucin

*L. salivarius* MP100 was strongly adhesive to both Caco-2 and HT-29 cell cultures. The mean ± SD number of adherent cells in 20 random microscopic fields was 351.4 ± 99.3 and 844.6 ± 137.8 in Caco-2 and HT29 cells, respectively. These values were similar to those achieved by *L. rhamnosus* GG (361.6 ± 108.9 and 820.2 ± 150.4, respectively). *L. salivarius* MP100 strongly adhered to porcine mucin since ~12.7% of the fluorescence was retained in the wells after the washing steps of the assay. This value was higher than that obtained for *L. rhamnosus* GG (9.57 ± 1.46).

### Safety Characterization of *L. salivarius* MP100

*L. salivarius* MP100 was susceptible to all antibiotics included within the EFSA criteria (27), with the exception of kanamycin (MIC: 128 μg/mL; EFSA cut-off value: 64 μg/mL) (Table 2). However, recent reports indicate that *L. salivarius* is intrinsically resistant to kanamycin (29, 51–54) due to lack of a transport system for this antibiotic (55). Moreover, *L. salivarius* MP100 was not hemolytic, was unable to degrade gastric mucin and did not produce biogenic amines *in vitro*.

**Table 2:** Minimum inhibitory concentrations (MICs) and cut-off values (μg/ml) for the antibiotics included within the EFSA criteria (27) for *L. salivarius* MP100.

**Antibiotics**	**Cut-off values**	**MICs (*L. salivarius* MP100)**
Ampicillin	4	0.5
Clindamycin	4	0.5
Chloramphenicol	4	2
Erythromycin	1	0.5
Streptomycin	64	32
Gentamicin	16	2
Kanamycin	64	128
Tetracycline	8	2
Vancomycin	n.r.	>128

*n.r., not required*.

### Suppression of Metaphylactic Antimicrobial Therapy in an Intensive Swine Farm With Replacement by Oral Administration of *L. salivarius* MP100

*L. salivarius* MP100 was administered (~9 log_10_ colony-forming units (CFU)/day) to pregnant sows (from the week before farrowing to the end of the lactation period) and to piglets (from day 12 after birth to day 15 after weaning) through an inoculated fermented feed (IFF). At the start of the IFF treatment, all antimicrobial supplementation was retired and only individual injectable treatments were applied when required. The treatment was systematically applied for a 2-year period.

The IFF administration of the putative probiotic strain led to substantial microbiological changes in the piglet feces over time. The total mean (±SD) microbial counts found by culture analysis in a subset of 8-week-old piglets tested before the start of the treatment (sampling time T1) was 7.05 (0.51) log_10_ CFU/g while in a similar subset, placed in the same box 2 years later (sampling time T2) it raised to 8.95 (0.30) log_10_ CFU/g. *L. reuteri* (formerly *L. reuteri*), *L. johnsonii* and *L. amylovorus* were the dominant *Lactobacillaceae* species found in the samples at T1 and T2. However, *L. salivarius* and *L. reuteri* counts significantly increased after 2 years of probiotic treatment (Table 3). Analysis by qPCR also revealed a significant increase of *L. salivarius* and *L. reuteri* DNA.

**Table 3:** Microbiological and biochemical parameters, expressed as mean (±SD), in the feces of 8-week-old piglets (*n* = 15) in the farm under study before (T1) and after 2 years (T2) of supplementation with *L. salivarius* MP100.

**Parameter**	**T1**	**T2**	***p*-value**
**Colony-forming units (log**_**10**_ **CFU/g)**			
Total *Lactobacillaceae*	7.05 (0.51)	8.95 (0.30)	<0.001
*L. salivarius*	2.63 (0.45)	4.30 (0.98)	0.006
*L. reuteri*	6.59 (0.50)	8.40 (0.33)	<0.001
*L. johnsonii*	5.28 (0.57)	5.14 (0.66)	0.603
*L. amylovorus*	4.66 (0.48)	4.57 (0.51)	0.692
**qPCR (DNA copies/g)**			
*L. salivarius*	2.58 (0.63)	4.14 (1.41)	0.031
*L. reuteri*	6.78 (0.62)	8.47 (0.45)	<0.001
**Short chain fatty acids (mg/g)**			
Butyrate	0.38 (0.06)	0.52 (0.05)	<0.001
Acetate	2.86 (0.14)	3.24 (0.25)	<0.001
Propionate	1.23 (0.05)	1.40 (0.05)	<0.001

The *L. reuteri, L. johnsonii*, and *L. amylovorus* isolates from T1 samples showed a high rate of antibiotic resistance, especially for tetracycline (100% for the *L. johnsonii* and *L. amylovorus* and >87.5% for the *L. reuteri* isolates) (Table 4). This last antibiotic was used in the farm under study until the sampling time T1. Interestingly, the antibiotic resistance rates sharply decreased after 2 years of antimicrobials withdrawal and concurrent probiotic treatment. In the case of tetracycline, it fell to 37.5, 25, and 12.5% for the *L. reuteri, L. johnsonii*, and *L. amylovorus* isolates, respectively. Similarly, the resistance rates for ampicillin, clindamycin, chloramphenicol and erythromycin dropped to 0% of the *L. reuteri* and *L. johnsonii* isolates (Table 4). The search for genes conferring resistance to tetracycline (*tetL, tetW*) and erythromycin (*ermB*), detected *ermB* and *tetW* in some T1 isolates. In contrast, none of these genes were harbored by the isolates pertaining to the T2 samples.

**Table 4:** Antibiotic resistance rates (%) among the *L. reuteri, L. johnsonii*, and *L. amylovorus* strains isolated from the feces of 8-week-old piglets in the study farm before (T1) and after 2 years of antibiotic withdrawal and supplementation with *L. salivarius* MP100 (T2).

	***L. reuteri***		***L. johnsonii***		***L. amylovorus***	
**Antibiotic**	**T1**	**T2**	**T1**	**T2**	**T1**	**T2**
Ampicillin	75	0	42	0	57	14
Clindamycin	25	0	42	0	62.5	12.5
Chloramphenicol	25	0	14	0	0	0
Erythromycin	50	0	12.5	0	75	12.5
Streptomycin	75	25	56	0	75	12.5
Gentamicin	75	25	84	25	12.5	12.5
Kanamycin^a^	100	100	100	100	50	25
Tetracycline	87.5	37.5	100	25	100	12.5
Vancomycin^a^	100	100	0	0	0	0

*The cut-off values were those established by EFSA (27)*.

a *L. reuteri and L. johnsonii are intrinsically resistant to kanamycin. L. reuteri is intrinsically resistant to vancomycin [assays for these antibiotics and species are not required by EFSA (27)]*.

The concentrations of SCFAs (acetate, propionate and butyrate) were highest in the feces of T2 piglets (Table 3). The differences between the T1 and T2 samples were statistically significant for all SCFAs.

The 16S rRNA gene-based metataxonomic analysis of the 45 fecal samples (15 from each group of piglets) yielded 4,606,781 high quality filtered sequences, ranging from 56,400 to 106,665 reads per sample [median (IQR) = 74,441 (70,811–84,339) sequences per sample]. The Shannon index median was 4.02 (3.70–4.21) (*p* < 0.001) and 4.05 (3.73–4.35) (*p* = 0.008) for the T1 and T2 samples, respectively.

**Figure 1:**
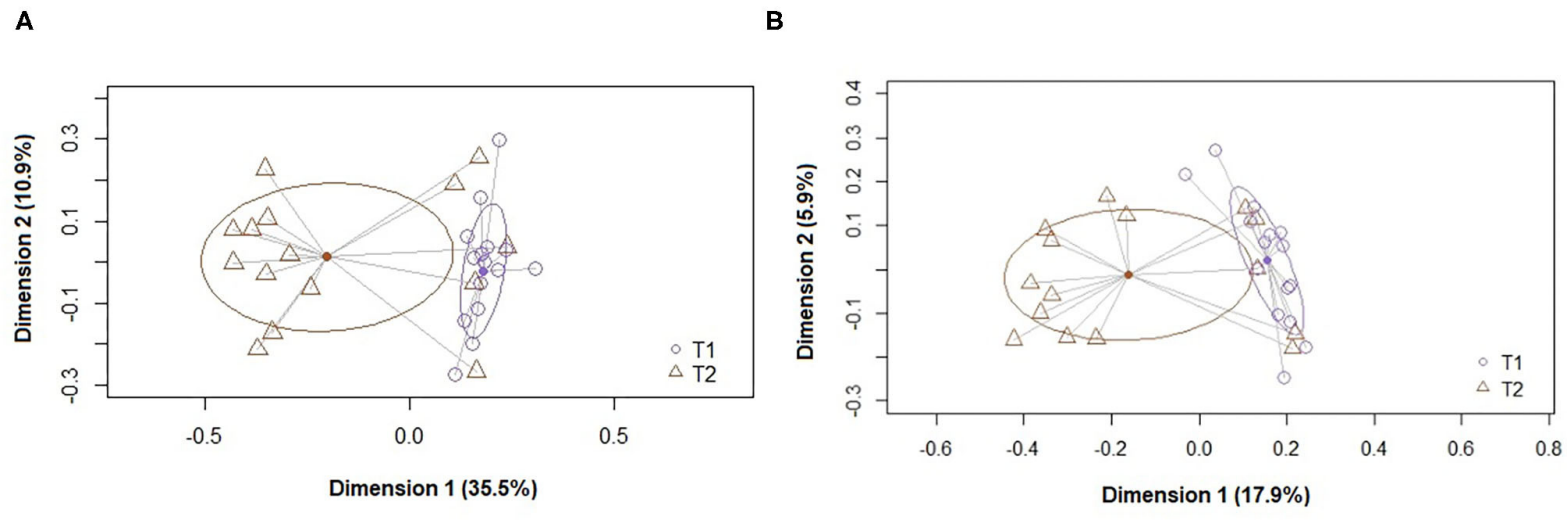
Principal coordinate analysis (PCoA) plots of bacterial profiles at the genus level based on **(A)** the Bray-Curtis dissimilarity analysis (relative abundance) and **(B)** the Jaccard's coefficient for binary data (presence or absence). The values on each axis label represent the percentage of the total variance explained by that axis. The differences between the groups of fecal samples (T1, T2) were analyzed using the PERMANOVA test with 999 permutations.

**Figure 2:**
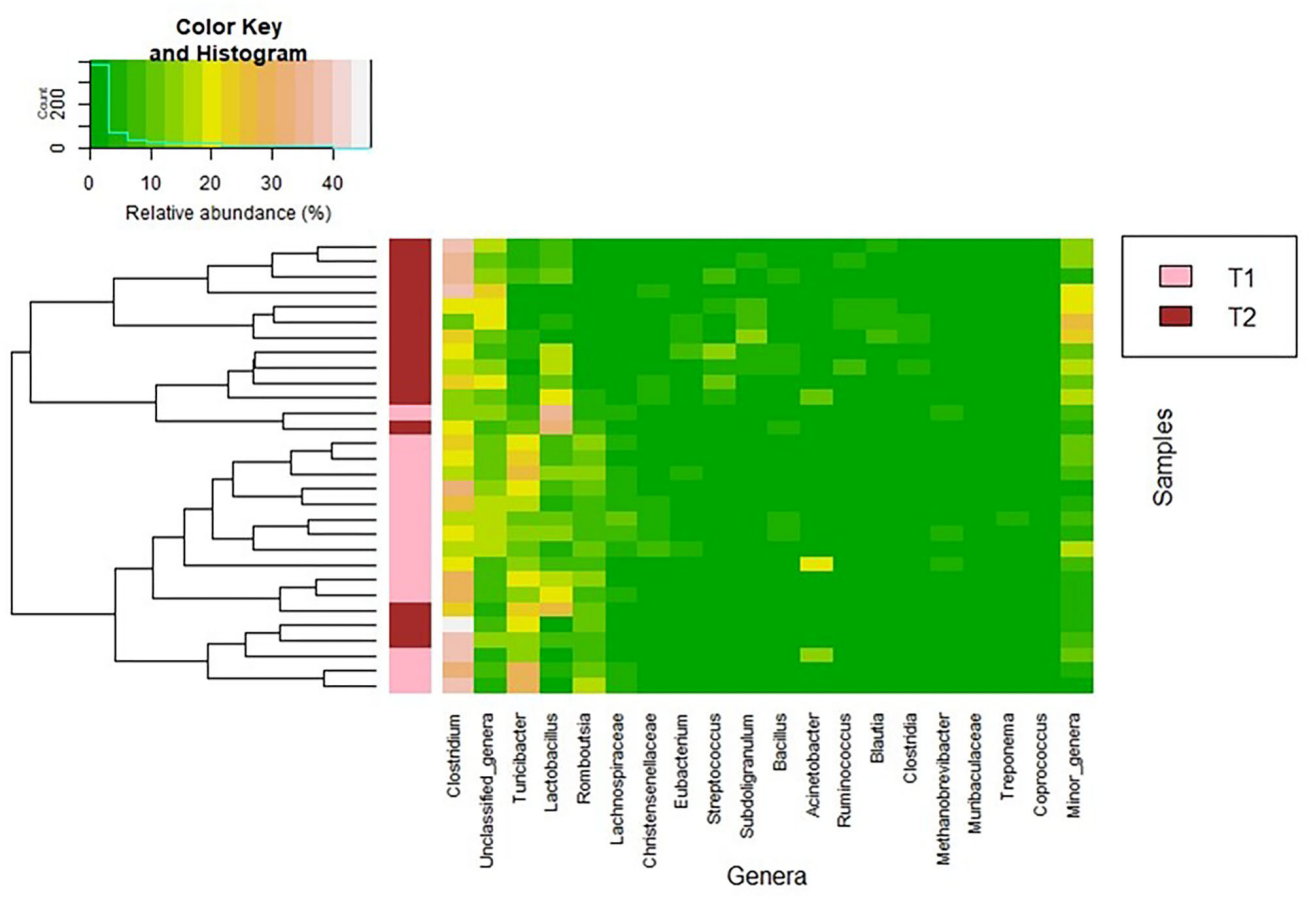
Heatmap showing the relative abundance of the 20 most abundant bacterial genera (x axis) detected in the fecal samples. The relative abundance of each bacterial genus within each sample is indicated by the color of the scale ranging from white (high relative abundance) to green (low relative abundance) as indicated in the scale shown at the left upper corner. Dendrogram linkages are based upon relative abundance of the genus within the samples and *hclust* was used as the clustering algorithm. The column between the dendrogram of the fecal samples and the individual values of the relative abundance of bacterial genera indicates the group of samples (T1 and T2).

At the ASV level, the PCoA plots of the Bray-Curtis distance matrix (abundance) revealed that most of the samples clustered according to their origin (T1 and T2) (Figures 1, 2). The subsequent pairwise analysis of similarity (PERMANOVA) revealed that the differences between the two sets of samples were statistically significant for all pairwise comparisons (*p* < 0.01). In the same way, differences were found in the Binnary Jaccard distance matrix (presence/absence) PCoA plot. Again, the samples clustered according to their origin (*p* < 0.01 for all pairwise comparisons) (Figures 1, 2).

Firmicutes was the most abundant phylum in both sampling times (median (IQR) relative abundance of 90.9% (89.88–95.13%) and 94.47% (92.64–95.78%), respectively; *p* = 0.348) (Table 5). At the genus level, *Clostridium* was the most abundant both in the T1 and T2 samples but its abundance increased from T1 to T2 samples (*p* < 0.001) (Table 5). Parallel, the relative abundance of *Turibacter, Romboutsia*, and *Lachnospiraceae* was higher in T1 samples than in T2 samples (*p* < 0.001). In addition, the relative abundance of the genera *Subdoligranulum, Ruminococcus*, and *Blautia* (which also contains several SCFAs-producing species) and, also, that of the genus *Streptococcus* were significantly higher in T2 than in T1 samples (Table 5). In relation to *Lactobacillus*-related sequences, its abundance increased from T1 to T2 samples (*p* =0.034).

**Table 5:** Relative frequencies, medians and interquartile ranges (IQR) of the relative abundance (%) of the most abundant bacterial phyla (in bold) and genera (in italics) detected in the T1 and T2 samples.

**Phylum/genus**	**T1**		**T2**		***p*-value**^**a**^
	***n***^**b**^ **(%)**	**Median (IQR)**	***n* (%)**	**Median (IQR)**	
**Firmicutes**	15 (100%)	90.9 (89.88–95.13)	15 (100%)	94.47 (92.64–95.78)	0.348
**Bacteroidota**	15 (100%)	2.04 (1.22–3.12)	15 (100%)	2.43 (1.50–3.59)	1.000
**Actinobacteriota**	15 (100%)	1.41 (0.74–2.12)	15 (100%)	1.20 (0.91–1.77)	1.000
**Proteobacteria**	15 (100%)	0.07 (0.04–0.33)	14 (93%)	0.03 (0.01–0.07)	0.110
**Euryarchaeota**	15 (100%)	1.44 (0.60–2.22)	13 (87%)	0.50 (0.13–0.85)	0.054
*Clostridium*	15 (100%)	12.07 (8.44–20.46)	15 (100%)	22.9 (19.23–36.66)	<0.001
*Lactobacillus*	15 (100%)	7.68 (2.79–13.34)	15 (100%)	8.84 (4.43–16.69)	0.034
*Turicibacter*	15 (100%)	18.42 (13.88–22.33)	15 (100%)	4.54 (2.83–8.03)	<0.001
*Romboutsia*	15 (100%)	10.63 (8.66–12.11)	15 (100%)	1.78 (1.06–4.80)	<0.001
*Subdoligranulum*	15 (100%)	0.13 (0.04–0.20)	14 (93%)	1.47 (0.41–5.32)	0.005
*Eubacterium*	15 (100%)	1.34 (0.86–2.24)	15 (100%)	2.67 (1.97–2.78)	0.056
*Lachnospiraceae*	15 (100%)	4.52 (3.11–4.88)	15 (100%)	0.86 (0.36–1.67)	<0.001
*Ruminococcus*	15 (100%)	0.33 (0.26–0.56)	15 (100%)	1.57 (0.43–3.11)	0.017
*Bacillus*	14 (93%)	0.74 (0.43–2.21)	15 (100%)	0.84 (0.43–3.54)	1.000
*Blautia*	15 (100%)	0.17 (0.06–0.26)	14 (93%)	0.80 (0.15–2.99)	0.044
*Streptococcus*	8 (53%)	0.01 (<0.01–0.03)	15 (100%)	0.51 (0.12–5.04)	<0.001
*Mogibacterium*	15 (100%)	0.38 (0.23–0.61)	15 (100%)	0.29 (0.19–0.58)	1.000
*Treponema*	15 (100%)	0.83 (0.41–1.11)	15 (100%)	0.31 (0.14–0.59)	0.180

a *Wilconson rank sum test*.

b *n (%): number of samples in which the phylum/genus was detected (relative frequency of detection)*.

## Discussion

In this work, the effects of the replacement of routine antimicrobial metaphylaxis by the oral administration of a putatively probiotic isolate of *L. salivarius* is described. The strain was obtained from the milk of a healthy sow with a record of reproductive excellence and was included in the feed of both sows and piglets, leading to a significant shift in the fecal metabolome and microbiota of 8 weeks-old piglets. The most relevant changes were the significant increases in the concentration of clostridia and the related SCFA metabolites (butyrate, acetate and propionate) that was accompanied by the improvement of practically all the productivity parameters associated to this farming. As expected, there also was a significant increase in the concentration of *Lactobacillus* related bacteria.

Weaning transition is one of the most critical periods in intensive swine farming (56). Piglets are weaned at an age in which they should still be consuming sow's milk for some additional weeks and, therefore, neither their intestinal tract nor their immune system are fully developed (57). At the same time, they have to adapt to very stressful conditions (maternal separation, changes in diet and environment, mixing with new mates), a fact that usually leads to a temporary reduction in the feed intake and a post-weaning growth retardation (58). As a result, they become an easy target for “nosocomial” microbes that are highly prevalent in intensive farming, including pathogenic bacteria causing sepsis, meningitis, arthritis and gastrointestinal diseases. This is the reason why antibiotics were used as growth promoters and, although the European Union has banned them, they are still widely and routinely used as metaphylactic or false therapeutic agents.

Several approaches have been proposed to improve gut health that might allow antibiotic use discontinuation. In this context, the gut microbiota is considered a key factor in swine's health, due to its metabolic (including feed conversion efficiency) and immune differentiation roles and its contribution in preserving the integrity of the intestinal barrier (59–61). As a consequence, the intestinal microbiota exerts a strong influence on sow productivity (62, 63). Some of the beneficial effects associated to the gut microbiota are the result of specific bacterial metabolites, such as SCFAs, including acetate, propionate, and butyrate. SCFAs play several roles in the gut, from primary source of energy to colonocytes, immunomodulation and protection against pathogens to biosynthesis of mucus, and water and mineral absorption (64–66).

Among SCFAs, butyrate has attracted most research attention because of its additional beneficial effects on animal production, including the improvement of growth performance (67–71). Butyrate is the main end product of some non-pathogenic species of the genus *Clostridium* (72–74), which contribute to preserve a healthy intestinal microecology through the control of the growth of pathogenic microbes (75–77), while living in harmony with commensal members of the families *Bacteroidaceae, Enterococcaceae*, and *Lactobacillaceae* (77). In a previous study, the relative abundance of SCFA-producing bacteria and the concentrations of acetate, propionate and butyrate were significantly higher among fecal samples from high-reproductive performance farms than among those from low-reproductive performance farms (78). Streptomycin treatment depleted butyrate-producing clostridia from the murine gut, decreased butyrate levels, and increased the population of pathogens like *Salmonella enterica* serovar Typhimurium (79). In contrast, direct administration of butyrate to pigs has shown to produce a variety of benefits to gut health, including the control of inflammation and the reinforcement of the barrier function (80, 81). Such positive effects of butyrate may depend on the age of the animals and, therefore, in-feed supplementation should be performed as early as possible in order to obtain better health outcomes (82). Overall, any strategy resulting in an increased production of SCFAs (and particularly butyrate) will have a relevant role in the post-antibiotic era of animal production.

In this work, the abundance of *Lactobacillus* and *Clostridium* sequences increased simultaneous. This observation is not strange since both kinds of microbes usually establish a collaborative network in the porcine's gut. Lactobacilli decrease the intestinal pH and produce lactic acid, which is required by clostridia to produce butyrate. In turn, it has been repeatedly observed that butyrate-producing clostridia have the ability to inhibit pathogenic bacteria in the intestinal tract while promoting the growth of lactobacilli (83–85). It has been shown that severe damage in the epithelium of the ileum mucosa of pigs experimentally infected with *Salmonella* Typhimurium was correlated with a decrease of *Lactobacillu*s and butyrate-producing anaerobic bacteria, including *Clostridium* spp. (86).

Lactobacilli are dominant bacteria in the pig gut microbiota during early life (87). Among the different *Lactobacillus* species, *L. reuteri* and *L. salivarius* are host-adapted species which share a long-term evolutionary history with swine (88, 89). In fact, they are among the few *Lactobacillus* species that can be isolated from mammalian milk, including sow's milk (14). As normal residents, this group of bacteria thus has an advantage over others in ecology for colonizing the gut. However, the normal process of acquisition of the piglet gut microbiota is greatly disrupted by the high social and physiological stress together with the abrupt interruption of the immune protection imposed by early weaning (90). Under such circumstances, the gut microbiota is characterized by a severe dysbiosis (91–93), with a sharp decrease of lactobacilli (93), and a high susceptibility to pathogen infection (94, 95). In this study, the administration of *L. salivarius* MP100 led to an increase of the *Lactobacillus* abundance. Culture-dependent analysis and species-specific detection of *L. salivarius* and *L. reuteri* by qPCR indicated that the increase in the *Lactobacillus* abundance was not due to a sharp increase in the concentration of *L. salivarius* (which was actually moderate) but to a notable increase in the population of *L. reuteri*. It has been previously shown that the administration of a probiotic strain may have a low impact in terms of colonization of that probiotic strain but a high impact in relation to the promotion of the growth of other beneficial members of the gut microbiota (96).

A large number of *in vivo* studies have assessed the impact of different probiotic *Lactobacillus* on the performance and health of weaned piglets (97). However, the results have been very heterogeneous depending on the tested probiotic product and the posology. This highlights the need for a better selection and characterization of the strains aimed to be used as swine probiotics. Anyway, some probiotics have successfully improved the health and performance in neonatal and growing pigs (98–100), including *L. reuteri* and *L. salivarius* strains (101–109).

The strategy followed in this work implied the administration of the probiotic strain to both sows and piglets. Previous studies have reported that providing sows and their piglets with the same strain simultaneously was more effective than feeding sows or piglets alone (21, 110–116).

As it has been stated above, both the use of butyrate and probiotics are usually considered among the potential candidates to substitute antibiotics. In this work, we have shown that the use of a well-characterized *L. salivarius* strain isolated from sow milk was able to drive a significant increase in the abundance of *Lactobacillus* and butyrate-producing clostridia, which resulted in significant increases in the concentration of the three assayed SCFAs, including butyrate.

This shift in the gut ecology of the treated animals was associated with a decrease in the prevalence of antibiotic-resistant lactobacilli. These microbes are good indicators of antibiotic pressure since they easily adapt to antibiotic-rich environments by different mechanisms, including the acquisition of transmissible genes (117, 118). This study shows that a 2-year period of antibiotic withdrawal is enough to reduce notably the burden of antibiotic resistances in a pig farm, a fact that must be highlighted in the frame of the current antibiotic resistance crisis. In this study a high percentage of *L. reuteri, L. johnsoni*, and *L. amylovorus* strains showed phenotypic resistance against many of the tested antibiotics. Antibiotic resistances among lactobacilli can be intrinsic [e.g., changes in the composition of the cell wall, as in the case of the intrinsic resistance of many *Lactobacillus* species to vancomycin (119)] or acquired through chromosomal mutations [e.g., a single mutation in the 23S rRNA gene reducing the affinity of erythromycin for the ribosome (120)]. The risk of inter-bacterial transfer of antibiotic resistances is insignificant for lactobacilli displaying intrinsic resistances or acquired resistances due to chromosomal mutations. In contrast, the potential of transmissible resistance genes (particularly those associated to mobile genetic elements) for horizontal spread is high and this risk deserves special attention because of its connotations for public health. In this context, several genes responsible for transmissible antibiotic resistance among lactobacilli have already been reported [reviewed in (118)].

Tetracycline resistance (*tet*) genes are the most common determinants of transmissible resistance in lactobacilli. It has been recently reported that the use of tetracycline selects the presence of transmissible genes conferring resistance not only to tetracycline (*tet* genes) but, also, to erythromycin (*erm* genes) in nursery pigs (121). As a consequence, such genes are widely spread in intensive pig farms using antibiotic metaphylactic approaches (122, 123). Presence of *tet* and *erm* genes seems to be relatively frequent among *L. reuteri* and *L. johnsonii* isolates from pork and poultry meat (124, 125), two of the farm sectors in which the use of antibiotics is particularly high. Sequencing of the *tetM* genes from such origin has revealed that there are almost identical (>99% sequence similarity) to *tetM* genes previously identified in human pathogens (*Neisseria meningitidis, Listeria monocytogenes*) (124). With respect to lactobacilli, it is long known that transference of transmissible antibiotic resistance can occur in different directions: (a) between different lactobacilli species/strains; (b) from lactobacilli to different Gram-positive bacteria, including relevant human pathogens (e.g., *Staphylococcus aureus*); and (c) from other Gram-positive bacteria to lactobacilli (126–128). In fact, the prevalence of *tet* and *erm* genes is very high among staphylococci (including *S. aureus* and *S. epidermidis*) isolates in the different steps of the chain of swine production, which pose a considerable risk for consumers (129). The presence of antibiotic selective pressure enhances the transfer of these resistance determinants (130). Use, abuse or misuse of antibiotics in intensive food production systems increases the chances of transmission of antibiotic resistant bacteria from livestock to humans (131–133). In addition, routine zinc supplementation in the swine diet has also been identified as a factor contributing to increase and maintain the presence of tetracycline resistance genes in the porcine gut (134–136).

Although lactobacilli are usually sensitive to β-lactamases and the *blaZ* gene has been rarely detected among these microbes (124), resistance to ampicillin was high among the strains isolated in this study. Future work will involve genome sequencing of those strains displaying high phenotypic resistance against this and other antibiotics and harboring the 3 resistance genes assayed in this work (*tetL, telW, ermB*).

Overall our study shows that the replacement of antibiotics by other microbiota-friendly approaches was feasible and led to positive microbiological and biochemical shifts in the enteric environment.

## Data Availability Statement

The datasets presented in this study can be found in online repositories. The data presented in the study are deposited in the NCBI Short Read Archive repository, accession number PRJNA705469.

## Ethics Statement

The animal study was reviewed and approved by Ethical Committee on Animal Experimentation of the Faculty of Veterinary of the Universidad Complutense de Madrid (Spain), protocol 33/17. Written informed consent for participation was not obtained from the owners because the owners (L'Albeitar) participated in the study and they are co-authors of this manuscript.

## Author Contributions

OS, PF, JM, AF, and JR designed and coordinated the study. OS, PF, JM, and AF designed the inoculated fermented feed. PF, JM, and AF administered the strain, collected the fecal samples and keep updated the farm database. RA, IP, and JR processed the samples and performed the microbiological and biochemical analyses. SD and LS analyzed the presence of antibiotic resistance genes among the lactobacilli strains. CA and LF executed bioinformatics and statistical analysis. JR drafted the manuscript. All authors contributed to the critical review of the manuscript and approved the final version submitted to this manuscript.

## Conflict of Interest

The authors declare that the research was conducted in the absence of any commercial or financial relationships that could be construed as a potential conflict of interest.
